# Polymicrobial odontogenic periorbital and orbital necrotizing fasciitis (PONF): A case report

**DOI:** 10.1016/j.ajoc.2022.101439

**Published:** 2022-02-18

**Authors:** Arman Mosenia, Abtin Shahlaee, Isaiah Giese, Bryan J. Winn

**Affiliations:** a Department of Ophthalmology, University of California San Francisco, 490 Illinois Street, San Francisco, CA, 94158, USA; b School of Medicine, University of California, San Francisco, 533 Parnassus Ave, San Francisco, CA, 94143, USA; c Department of Ophthalmology, California Pacific Medical Center, 711 Van Ness Avenue, San Francisco, CA, 94102, USA; d San Francisco Veterans Affairs Medical Center, 4150 Clement Street, San Francisco, CA, 94121, USA

**Keywords:** Odontogenic, Periorbital necrotizing fasciitis, Orbital necrotizing fasciitis, Polymicrobial, *Streptococcus milleri* group, *Staphylococcus lugdunensis*, Microbiology

## Abstract

**Purpose:**

To present a case of periorbital and orbital necrotizing fasciitis (PONF) from an odontogenic source with a distinct microbiologic profile and highlight the need for emergent multidisciplinary management.

**Observations:**

A 39-year-old man presented with periorbital swelling, pain, and erythema following facial trauma. Imaging revealed peri-dental collections, accompanying maxillary sinusitis, and pre- and post-septal involvement. Immediate surgical debridement of necrotic tissue along with broad-spectrum antibiotics were pursued for management. Cultures grew multiple organisms, most notably *Streptococcus milleri* group and *Staphylococcus lugdunensis*.

**Conclusions and Importance:**

PONF is a rare yet potentially fatal disease. *Streptococcus milleri* group and a fulminant course are to be suspected when the source is odontogenic. Timely multidisciplinary surgical debridement and medical management with intravenous antibiotics is critical for best outcomes.

## Highlights

• Odontogenic cases of periorbital and orbital necrotizing fasciitis (PONF) may have a fulminant course.
• *Streptococcus milleri group* is associated with odontogenic PONF.
• An emergent multi-disciplinary surgical and medical approach to PONF is highly recommended.
• Antibiotics may be optimized based on known virulence factors of suspected organisms.

## 1 Introduction

Necrotizing fasciitis (NF), characterized by necrosis of subcutaneous tissue and fascia, is a progressive and potentially fatal soft tissue infection. Periorbital/orbital NF (PONF) is rare, with an incidence of 0.24 cases per million per year.^1^ Risk factors include rheumatologic diseases, immunosuppression, alcoholism, and diabetes mellitus,^2^ and it is frequently preceded by trauma or surgery. Here, we present a unique case of odontogenic PONF following trauma with a distinct microbiological profile and highlight the importance of immediate multidisciplinary care.

## 2 Case report

**Fig. 1:**
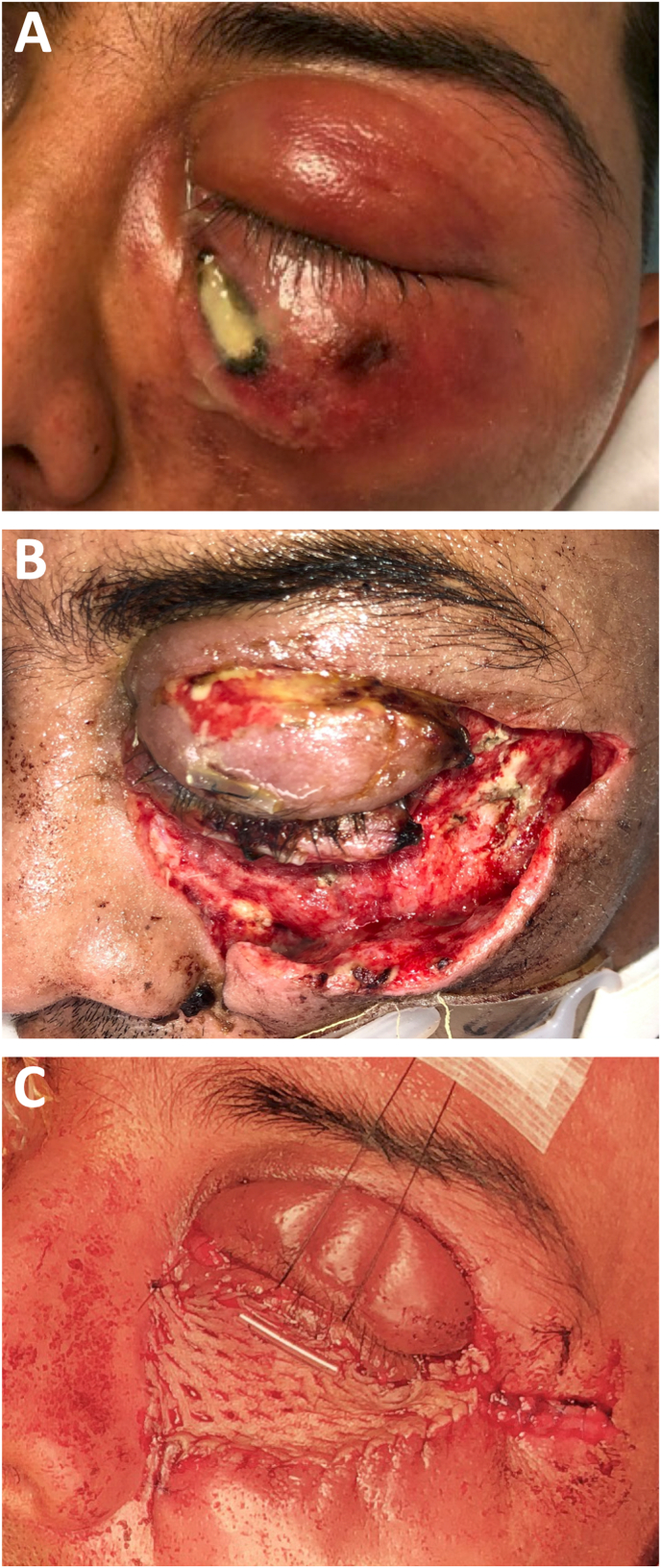
A) Initial presentation demonstrating prominent periorbital swelling and purulent discharge with skin necrosis B) Status post three rounds of exploration and debridement C) After reconstruction of defects at 2 weeks with split-thickness skin graft and Frost suture tarsorrhaphy.

**Fig. 2:**
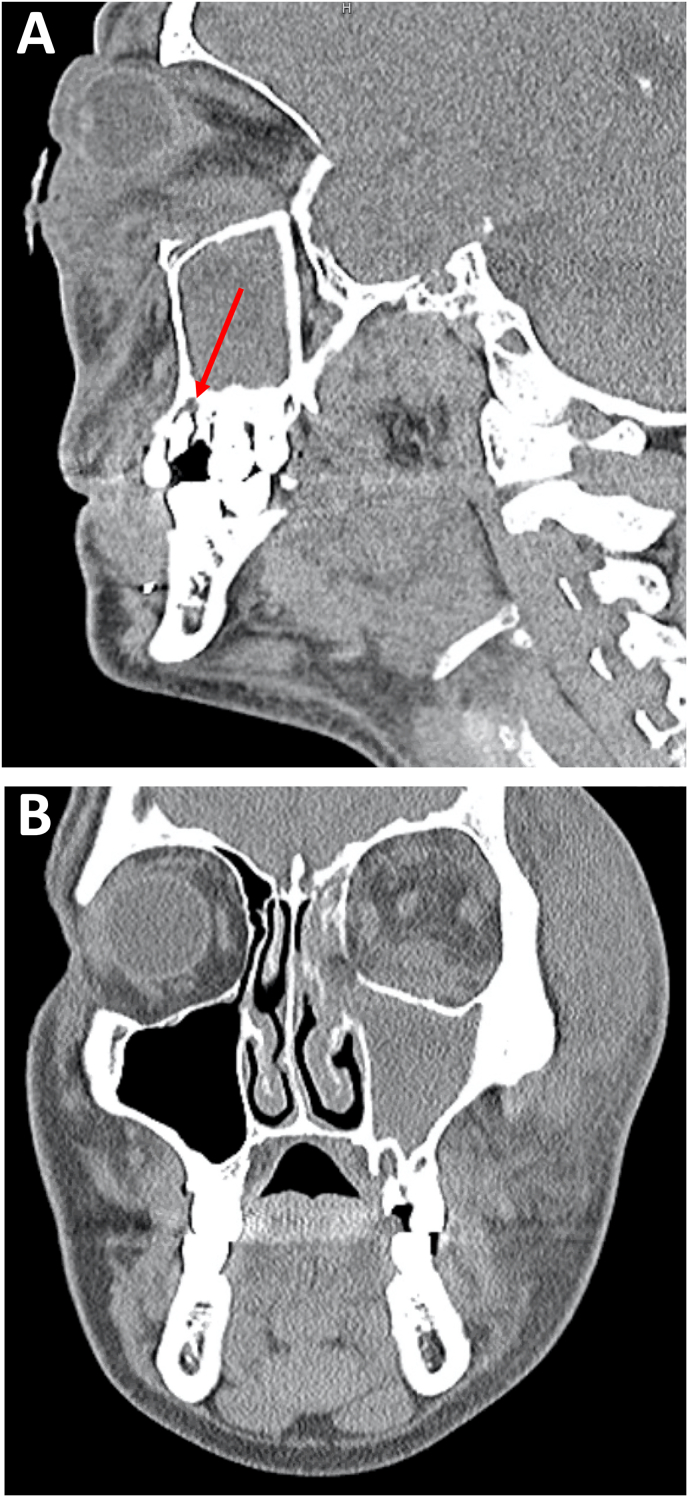
Non-contrast CT scans at initial presentation: A) sagittal view illustrating peri-dental lucency surrounding the first upper left molar tooth (arrow). B) Maxillary sinusitis, and significant pre- and post-septal collections on the left side concerning for necrotizing fasciitis on coronal view.

A 39-year-old man was referred to the emergency department with progressive left-sided periorbital swelling, erythema, and pain following blunt facial trauma two days earlier. His medical history was only remarkable for heavy alcohol use. Initial ophthalmic evaluation revealed left-sided proptosis, relative afferent pupillary defect, and diffuse restriction of extraocular motility, especially in supraduction. In the affected eye, visual acuity was limited to 20/200 and intraocular pressure (IOP) was 17 mmHg. Vision and IOP were 20/25 and 8 mmHg in the fellow eye, respectively. The left lower eyelid was tense and demonstrated an area of obvious skin necrosis and purulent discharge medially (Fig. 1A). C-reactive protein (186 mg/L) and white blood cell count (11.2 × 10^9^/L) were elevated, sodium (132 mmol/L) was decreased, and hemoglobin (15.6 g/dL), non-fasting glucose (116 mg/dL), and creatinine (0.66 mg/dL) were normal. Intravenous clindamycin, vancomycin, and piperacillin-tazobactam were initiated. Non-contrast CT scan was notable for comminuted bilateral nasal bone and septum fractures, peri-dental lucency surrounding the first upper left molar tooth with concomitant maxillary sinusitis, and significant pre- and post-septal collections (Fig. 2). The patient was taken emergently to the operating room for exploration. Profuse purulent discharge and avascular tissue were encountered and debrided in the left upper and lower eyelids and cheek as well as anterior and intraconal orbit consistent with necrotizing fasciitis. Concomitant maxillary antrostomy, ethmoidectomy, and extraction of the infected tooth were performed. Re-exploration and limited debridement were performed 12 hours later. Dilated fundus examination revealed ischemic retina with serous retinal detachment over the posterior pole and central folds over the macula. The area was again explored 48 hours after initial surgery without any evidence of additional necrosis. Intraoperative cultures grew moderate oronasal flora, gram positive anaerobic bacteria, numerous polymorphic bacteria from the *Streptococcus milleri group,* and few *Staphylococcus lugdunensis*. Antibiotics were narrowed to intravenous ceftriaxone and metronidazole and were continued for 7 days. Visual acuity was diminished to no light perception on the left. At 2 weeks, the patient underwent a repair of the left lower lid and cheek defects with a split thickness skin graft (Fig. 1) due to limited vascular supply in the heavily debrided area and risk of full-thickness skin graft failure. Six-months post-operatively, the patient had expectedly developed cicatricial ectropion of the lower lid with anterior lamella shortening, which was successfully repaired.

## 3 Discussion

Cases of NF originating from an odontogenic infection are infrequent and mostly reported in the context of lower head and neck involvement. PONF secondary to an odontogenic source has only been reported in three instances.^3^, ^4^, ^5^ These cases were triggered by tooth extraction or an isolated tooth infection. In our case, there was a tooth infection along with periocular trauma, both of which are individually associated with NF^2^ and may have had synergistic effects. Heavy alcohol use has also been previously linked to PONF^2^ and could have contributed to the risk of developing NF in this patient.

The most common organisms in PONF are mixed flora (including anaerobic bacteria), group A beta-hemolytic *Streptococcus*, *Pseudomonas aerogenosa*, and *Staphylococcus aureus* (including methicillin-resistant *Staphylococcus aureus*).^2^ Compared to NF elsewhere, PONF may evolve rapidly and involve adjacent cervical, thoracic, and intracranial areas. Fibrofatty tissue invasion via enzymes and toxins that are produced by these bacteria, including hyaluronidases, lipases, collagenases, and steptokinase, facilitate their rapid spread. Consequently, mortality directly correlates with time to intervention. The first major clinical decision point at presentation is to distinguish preseptal or orbital cellulitis from PONF. Calculating the Laboratory Risk Indicator for Necrotizing Fasciitis (LRINEC)^6^ score can aid in this decision (Table 1). Scores of 6 or more (our patient's score was 6) indicate a high likelihood of necrotizing fasciitis. In addition to emergent surgical management, immediate broad-spectrum antibiotic therapy with a combination of vancomycin and a beta-lactam (piperacillin-tazobactam) was used to cover the most common organisms. Clindamycin, a protein synthesis inhibitor, was added for its ability to directly neutralize DNase, steptolysin O, and streptococcal pyrogenic exotoxins.^7^^,^^8^

**Table 1:** LRINEC Score for necrotizing soft tissue infection is a useful tool for evaluating PONF. A score of 6, as seen in this case, indicates an intermediate risk for NF and has a positive predictive value of 92% and negative predictive value of 96%.^6^ Adapted from Wong C–H, Khin L-W, Heng K–S, Tan K–C, Low C–O. The LRINEC (Laboratory Risk Indicator for Necrotizing Fasciitis) score: a tool for distinguishing necrotizing fasciitis from other soft tissue infections. *Crit Care Med*. 2004; 32 (7):1535–1541.

Patient's Score	LRINEC Variable	Score
	C-Reactive Protein (mg/L)	
+4	< 150	0
(186 mg/L)	≥ 150	4
	Total white cells (10^9^/L)	
0	< 15	0
(11.2 × 10^9^/L)	15–25	1
	> 25	2
	Hemoglobin (g/dL)	
0	≥ 13.5	0
(15.6 g/dL)	11–13.5	1
	< 11	2
	Sodium (mmol/L)	
+0	≥ 135	0
(132 mmol/L)	< 135	2
	Creatinine (mg/dL)	
+0	≤ 1.6	0
(0.66 mg/dL)	> 1.6	2
	Glucose (mmol/L)	
+0	≤ 180	0
(132 mmol/L)	> 180	1
6	Total	13

Intraoperative cultures were positive for oronasal flora, gram positive anaerobes, *Staphylococcus epidermidis,* and most notability *Streptococcus milleri* group and *Staphylococcus lugdunensis*. *S. lugdunensis* has been reported to cause destructive infections in the head and neck region^9^ and is frequently isolated from oral collections.^10^ Despite its virulence factors, including delta-toxin-like activity, resistance to lysosomes, and biofilm formation,^11^ *S. lugdunensis* is generally susceptible to a wide variety of antibiotics. To date, only one case of PONF with this organism has been reported, which was mono-bacterial.^2^ Polymicrobial NF in the periorbital/orbital region is uncommon and often associated with significantly higher mortality,^2^ and our case serves as the first reported polymicrobial case of PONF involving *S. lugdunensis*.

*Streptococcus milleri* group, also known as *Streptococcus anginosus* group, has been reported in orbital cellulitis,^12^^,^^13^ but only a few cases of PONF.^3^, ^4^, ^5^ While infection by members of this group of bacteria may arise in a variety of body sites^14^*, S. milleri* group has been present in all three previous cases of odontogenic PONF.^3^, ^4^, ^5^ These organisms have a polysaccharide capsule, form biofilms, and have a tendency to adhere to extracellular matrix components such as fibronectin via cell surface proteins.^15^^,^^16^ Given that all reported odontogenic cases have documented positive cultures for this group of bacteria, providers should anticipate the presence of this organism and a fulminant clinical course in odontogenic PONF.

## 4 Conclusions

We present a case of PONF with a unique presentation and combination of organisms highlighting the need for emergent surgical and multidisciplinary management. *Streptococcus milleri* group is associated with odontogenic PONF. Both *S. milleri* and *S. lugdunensis* likely contributed to PONF in this case. Clinicians should anticipate a fulminant course of disease especially when the underlying infection is odontogenic and polymicrobial.

## Patient consent

Written informed consent was obtained from the patient for publication of this case report and accompanying images. A copy of the written consent is available for review by the Editor-in-Chief of this journal on request.

## Funding

This manuscript was supported, in part, by the 10.13039/100008069UCSF Vision Shared Resource Core Grant (10.13039/100000053NIH/NEI P30 EY002162) and departmental support from 10.13039/100001818Research to Prevent Blindness.

## Authorship

All authors meet the ICMJE criteria and have significantly contributed to the creation of this manuscript.

## Declaration of competing interest

The authors declare that they have no known competing financial interests or personal relationships that could have appeared to influence the work reported in this paper.
